# Multiple esophageal hematomas with oozing observed and successfully treated after transcatheter mitral valve edge‐to‐edge repair procedure

**DOI:** 10.1002/deo2.75

**Published:** 2021-11-24

**Authors:** Yasuyuki Shirai, Yoshihiro Kinoshita, Tatsuya Koumoto, Michitaka Kawano, Ayako Ogoshi, Katsunori Harada, Akihiro Isotani, Shinichi Shirai, Kenji Ando, Tomoharu Yoshida

**Affiliations:** ^1^ Department of Gastroenterology Kokura Memorial Hospital Fukuoka Japan; ^2^ Department of Cardiology Kokura Memorial Hospital Fukuoka Japan; ^3^ Kitakyushu Kokura Hospital Fukuoka Japan

**Keywords:** esophageal mucosa, esophagogastroduodenoscopy, hematoma, mitral valve insufficiency, transesophageal echocardiography

## Abstract

Although previously reported as relatively rare, esophageal hematoma can likely develop in patients on anticoagulants or those with underlying hemorrhagic disorders. From April 2018 to December 2018, among 36 patients who received transcatheter mitral valve edge‐to‐edge repair (TMVr) at our hospital, seven (19.4%), who were suspected of having digestive tract hemorrhage evidenced by blood stains on a probe extracted after transesophageal echocardiography, underwent esophagogastroduodenoscopy. Esophageal hematomas were noted in all patients, and endoscopic hemostasis was performed in two cases. Depending on their form, hematomas were noted on the submucosa and the epithelium of the shallow esophageal layer. Esophageal hematomas caused by transesophageal echocardiography for TMVr are not rare, and clinicians should be aware of it.

## INTRODUCTION

Esophageal hematoma was reported in 1957 as an illness involving hematoma formation on the esophageal submucosa. Its causative factors include mechanical and idiopathic injury, and it is likely to develop in patients on antithrombotic drugs or those with underlying hemorrhagic illnesses.^1^, ^2^, ^3^ Transcatheter mitral valve edge‐to‐edge repair (TMVr) is a catheter therapy for mitral valve incompetence. TMVr clips the two leaflets of the mitral valve together and helps in valve closure. It is performed concurrently with transesophageal echocardiography (TEE) that examines the mitral valve. Compared with other TEE‐related treatments, TMVr requires several hours of TEE. Moreover, esophageal perforation, laceration, and hematoma have been reported in TEE‐related treatment.^4^ Herein, we report cases of esophageal hematomas caused by TEE for TMVr.

## CASE REPORT

Since its induction at the Cardiology Department of this hospital in April 2018, 36 patients have undergone TMVr as of December 2018. MitraClip (Abbott Medical Japan LLC, Tokyo, Japan) was used for TMVr. The TEE probe X8‐2t (Philips Medical Systems, Andover, MA, USA) was used. Antithrombotic drugs were administered to all patients; anticoagulants (warfarin, direct oral anticoagulant) were orally administered to 22 patients; antiplatelet drugs (aspirin, clopidogrel) to eight patients; and anticoagulant and antiplatelet drugs co‐administered to six patients. Antithrombotic drugs were continued, and on the day of treatment, these regimens were followed by intravenous administration of 5000 IU of heparin. Immediately after TMVr completion, seven (19.4%) patients suspected of having digestive tract hemorrhage, evidenced by the slightest amount of blood stain on a probe extracted after TEE, underwent esophagogastroduodenoscopy (EGD). TMVr treatment was performed under general anesthesia. EGD was conducted before or immediately after extubation.

A blackish brown protuberance associated with hemispherical hematoma was noted in all seven patients undergoing EGD. Two patients showed only one, whereas five patients showed several hematomas. The sites were the throat in two patients, upper esophagus in six, middle esophagus in four, and lower esophagus in one patient (sites overlapped in two or more areas in some patients). Oozing was noted in two patients, who were treated with ligation with O‐ring and hemostasis with clipping. Patients without esophageal hematoma fasted only on the day of treatment, and those with esophageal hematoma fasted for 2–3 days followed by proton pump inhibitors (PPIs) administration. The two patients treated with hemostasis showed hematoma augmentation on the day of treatment and a day after; however, the hematoma shrank and disappeared thereafter. Four patients who underwent EGD continued to show hematoma shrinkage tendency within 3 days without hematoma resorption. Hematoma disappeared in two patients observed after 6 days (Table 1). No other complications, including perforation or Mallory–Weiss syndrome, were noted. In all cases, the clinical course was favorable with no hemorrhage noted the following day onward. Among the seven cases, we report two cases in particular as the esophageal hematoma shape in case 1 suggested hematoma presence in the epithelium and the submucosa, whereas in case 2, the progress of the hematoma was typical and followed up by endoscopy.

**TABLE 1:** Characteristics and clinical course of patients who developed esophageal hematoma

Case	Age	Sex	Part of the esophagus	Single or multiple	Follow‐up of endoscopy	Endoscopic procedure
1	73	Male	Middle	Single	—	—
2	82	Male	Upper	Multiple	Slightly shrank on the following day. Persisted 2 days later but almost improved 6 days later.	—
3	79	Female	Upper, middle	Multiple	—	—
4	74	Male	Upper, middle	Multiple	—	—
5	85	Female	Pharynx, upper	Multiple	Augmented on the following day. Showed a tendency to shrink 3 days later. Disappeared 6 days later.	Clip
6	75	Female	Upper	Single	Persisted 1 day later. Showed a tendency to shrink 3 days later.	—
7	84	Male	Pharynx, upper, middle, lower	Multiple	Showed a tendency toward improvement 2 days later. Disappeared 7 days later.	Endoscopic band ligation

### Case 1

**FIGURE 1:**
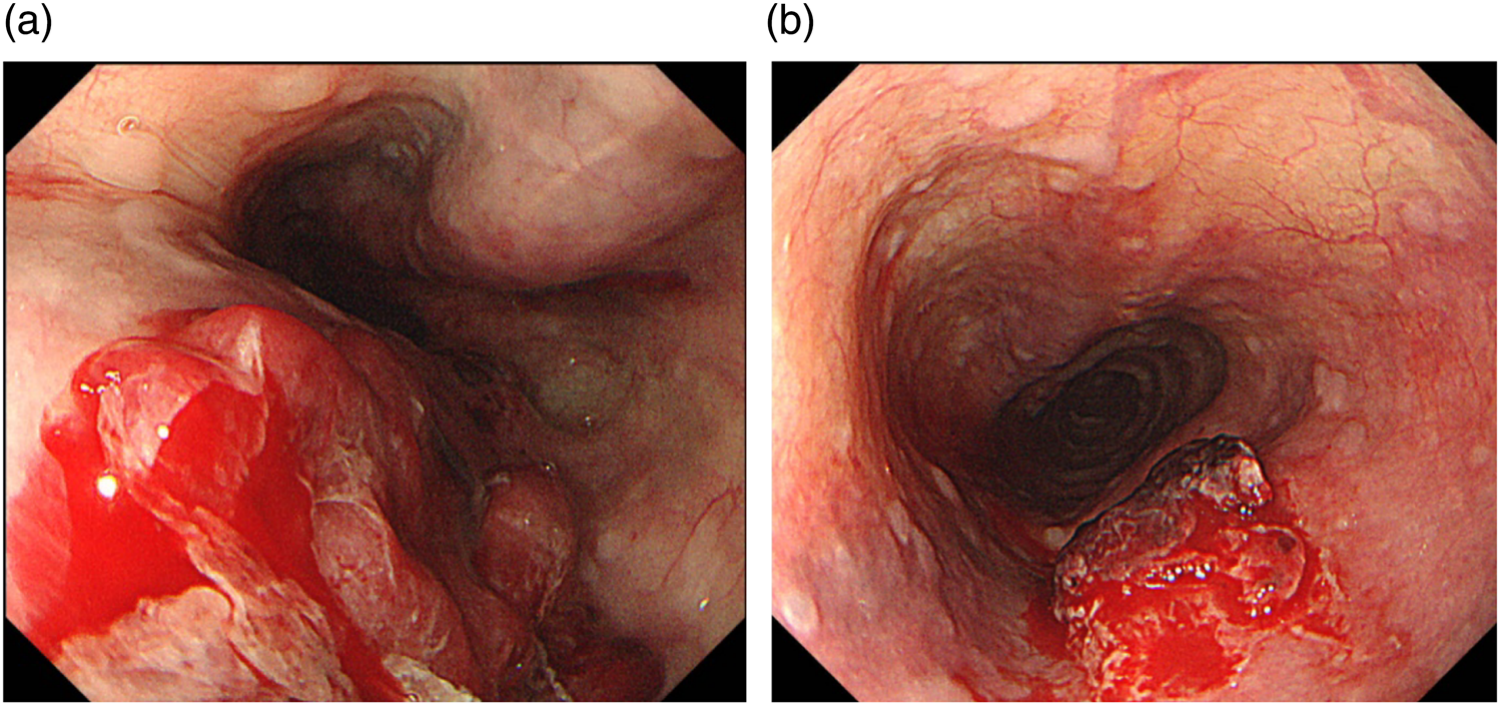
Multiple hematomas in the upper esophagus. (a) Esophagogastroduodenoscopy (EGD) immediately after transcatheter mitral valve edge‐to‐edge repair (TMVr). Multiple sites featured smooth‐surfaced, gently rising protuberances. The hematoma at the 1–2‐o'clock direction exhibited a dendritic vascular plexus. A hematoma was present at the 5–9‐o'clock direction. A hemispherical hematoma was formed on the surface, and it was hemorrhagic. (b) EGD on the following day. Blood remained on the surface partially, but the hematoma itself was significantly reduced

An 82‐year‐old male with underlying mitral valve incompetence and atrial fibrillation was on warfarin. After TMVr, a blood stain was noted on a TEE probe, and thus, EGD was conducted before extubation. Multiple hematoma sites were noted in the upper esophagus (Figure 1). A hematoma on the right wall showed a dendritic vascular plexus on the surface. On the left wall, a hematoma developed over another hematoma. The epithelium that developed on the surface of the hematoma was thin, which tended to bleed with insufflation or a subtle touch, presenting with collapsing lesions. This was treated with PPI administration and 2 days of fasting. On the following day, the hematoma shrank significantly.

### Case 2

**FIGURE 2:**
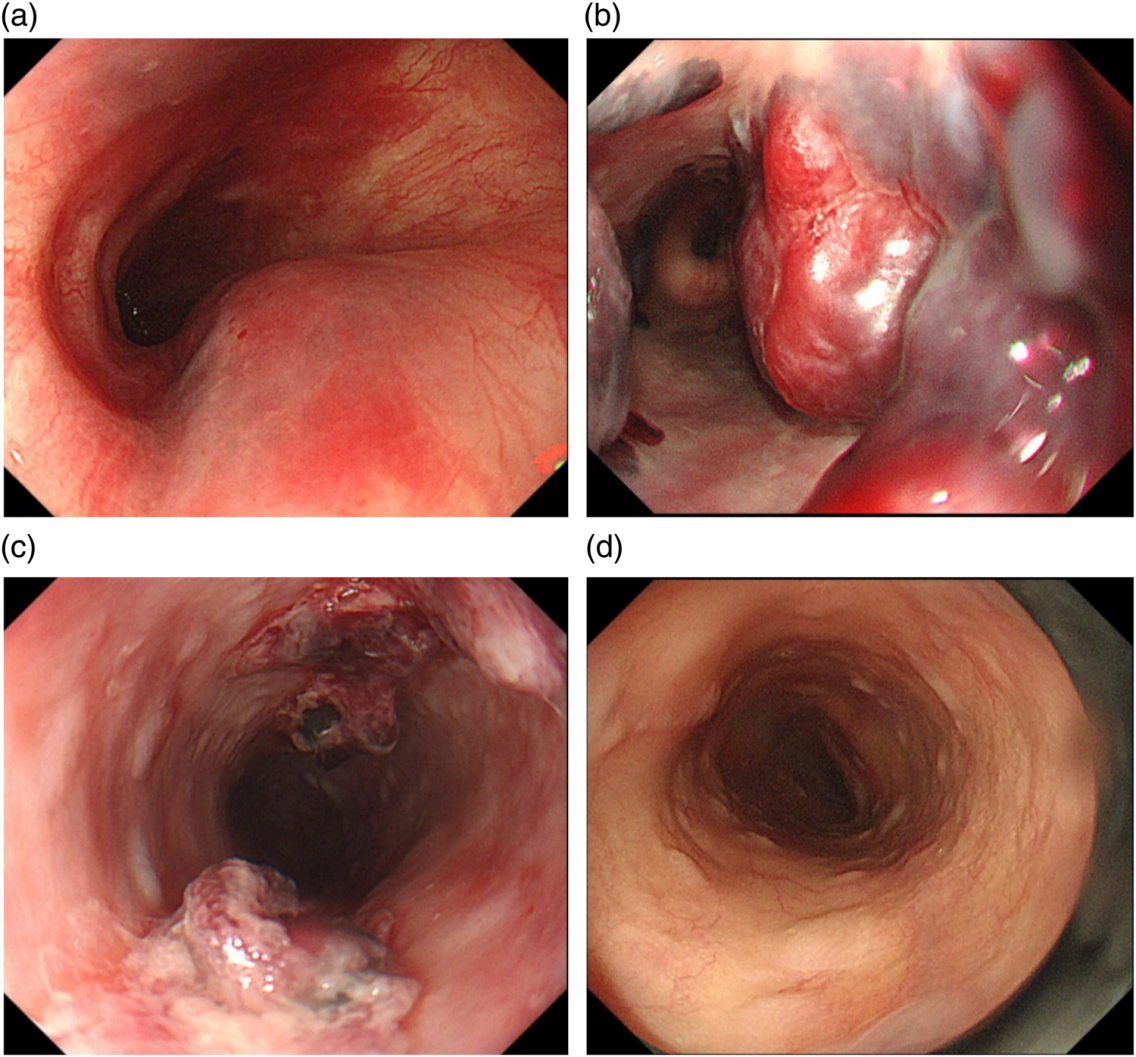
Change in the form of esophageal hematoma with time. (a) Esophagogastroduodenoscopy (EGD) immediately after the treatment with transcatheter mitral valve edge‐to‐edge repair (TMVr). A papule was present, gently rising and dominating one third of the lumen. Dendritic vessels were visible on the surface. (b) Hemostasis occurred 3 h later, and EGD was performed. Multiple sites featured dark red hematomas. Endoscopic band ligation (EBL) was conducted on the oozing sites. (c) EGD 2 days later. Post‐EBL ulcers were present, but the hematoma almost resolved. (d) EGD 7 days later. The hematoma did not form an ulcer and was resorbed

An 84‐year‐old male with underlying mitral valve incompetence, myocardial infarction (drug‐eluting stent placement), chronic cardiac failure, nonsustained ventricular tachycardia, chronic kidney disease, diabetes mellitus, and cerebral infarction was on antithrombotic drugs (aspirin and warfarin). After TMVr, a blood stain was noted on the TEE probe; thus, EGD was conducted. Despite the presence of a hematoma where gently rising dendritic vessels were visible, we terminated EGD after observation. Hematemesis was observed after 3 h, and EGD was performed again. Hematoma augmentation was observed at multiple sites from the throat to all areas of the esophagus with some oozing. Thus, endoscopic ligation of two bands was conducted. PPI administration and a 3‐day fasting period resulted in hematoma shrinkage 2 days later and in resorption 7 days later. The patient experienced nausea during the clinical course but reported no chest pain (Figure 2).

## DISCUSSION

Esophageal hematomas due to TEE‐related procedures have been reported^1^, ^2^; however, reports on esophageal hematoma caused by TEE for TMVr in recent years are few.^5^, ^6^ Because TEE for TMVr requires longer procedure time and aggressive use of TEE in the easily bleeding state during antithrombotic medication compared with other TEE‐related procedures, it reportedly poses a higher risk for complications.^4^ The probe must be carefully manipulated, and rough movement of the probe must be avoided during angulation. Shortening the time required to do so may reduce esophageal hematoma. This report is based on data from the early phase of TMVr. We believe that the frequency of hematomas has reduced owing to careful endoscopy procedures and less time required for performing them in recent times. Previous reports revealed that chest pain, dysphagia, and hemorrhage cause esophageal hematomas, and these hematomas spread extensively from the upper to the lower esophagus.^5^, ^7^, ^8^, ^9^ Moreover, an ulcer was formed in the process, but resolved on treatment after a few weeks.^7^, ^9^ In the present cases, hemispherical and relatively small hematomas tended to develop at multiple sites. This was possibly because we actively selected mild cases showing a trace of blood on the TEE probe and performed endoscopy immediately after. These hematomas showed a shrinking tendency within a few days and resolved a week later without scarring. If EGD is conducted for all patients immediately after TMVr installation, hematomas may be identified more frequently.

Excessive hemorrhage requiring hemostasis using a Sengstaken–Blackmore tube has been reported^7^; however, it can be conservatively treated in many cases through fasting for a few days after onset along with fluid administration and PPI use. No consensus has been reported on the criteria, methods, and follow‐up for endoscopic hemostasis. Because the hematoma was small, we chose a clip and endoscopic band ligation (EBL). Using EBL and clips for large hematomas, as previously reported, may worsen the bleeding by damaging the epithelium. If the hemorrhage is mild and multiple in number, using hemostatic agents without endoscopic hemostasis can be considered. We performed endoscopic hemostasis in two patients, but in hindsight, bleeding occurred because of ruptured hematoma, and natural hemostasis was more likely to ensue as the hematomas collapsed, and heparin effect wore off. In our experience, relatively small esophageal hematomas do not rebleed after a few days of fasting. The duration of fasting should be considered based on the hematoma status.

Till date, esophageal hematoma has been referred to using different names, including esophageal hematoma,^1^ dissecting intramural hematoma,^2^ intramural hematoma of the esophagus,^7^ esophageal submucosal hematoma,^8^ intramural esophageal dissection.^9^ These terms refer to the same illness of submucosal hematoma. As for the esophageal structure, vessels going through the muscular layer form a dendritic vascular plexus around the muscular layer of the mucosa.^10^ The endoscopic images of cases with dendritic vascular plexus on the surface showed deep hematomas, primarily in the submucosa. Conversely, case 2 (Table 1) showed a hemispherical, strong reddish hematoma without dendritic vessels, steeply rising on another hematoma. These are considered hematomas in the mucosal epithelium or directly beneath the epithelium. For a definite diagnosis of a peeled site, biopsy and endoscopic mucosal resection results may serve as a good reference. However, we did not perform biopsies as refraining from stimulating hematomas developed from iatrogenic causes in extremely hemorrhagic conditions is prudent. Further accumulation and discussion of cases will be necessary going forward with regard to the sites of hematomas.

In conclusion, active EGD after TMVr resulted in highly frequent identification of esophageal hematoma. In some patients, esophageal hematoma appeared to have developed not only on the submucosa but also on the mucosal epithelium.

## CONFLICT OF INTEREST

Shinichi Shirai was supported by Edwards Life Science and Medtronic as TAVI clinical proctor, Abbott as speaker honorary and MitraClip training faculty. Kenji Ando was supported by Terumo, Japan Lifeline, Medtronic Japan as lecture fees. Other authors have no conflict of interest to declare.

## FUNDING INFORMATION

None

## ETHICS APPROVAL AND CONSENT TO PARTICIPATE

The present study conforms to the Declaration of Helsinki (As revised in Fortaleza, Brazil, October 2013). The patients provided written informed consent before the procedures.

## Data Availability

Relevant data are available within the article.
